# A187 VANCOMYCIN CAN INDUCE AND MAINTAIN REMISSION OF REFRACTORY INFLAMMATORY BOWEL DISEASE IN PATIENTS WITH PRIMARY SCLEROSING CHOLANGITIS – A CASE SERIES

**DOI:** 10.1093/jcag/gwae059.187

**Published:** 2025-02-10

**Authors:** A Cintosun, N Narula

**Affiliations:** McMaster University, Hamilton, ON, Canada; McMaster University, Hamilton, ON, Canada

## Abstract

**Background:**

The microbiome is suspected to play a role in the pathogenesis of inflammatory bowel disease (IBD) and primary sclerosing cholangitis (PSC). Meta-analyses have shown a positive response for antimicrobials in IBD, although the results are difficult to apply clinically given the wide variability in antimicrobial modality and treatment regimen as well as perceived reduced effectiveness compared to advanced therapies. Fewer antimicrobial studies have been performed in the PSC population. There is some evidence that vancomycin may represent a treatment option with concurrent IBD and PSC.

**Aims:**

To determine the clinical and endoscopic effect of vancomycin for patients with refractory PSC-IBD.

**Methods:**

Retrospective chart review.

**Results:**

We identified 6 patients (4 male) with IBD and PSC who were started on vancomycin (Figure 1). Four had ulcerative colitis (UC) and 2 had Crohn’s disease. Three had pre-existing cirrhosis; 1 of these patients had undergone liver transplantation for PSC. All had failed multiple therapies for IBD with up to 4 advanced therapies failed. One patient had previously required surgery for UC.

Patients were started on induction vancomycin at 125 mg 4 times daily or BID (twice daily) for 8 weeks followed by maintenance therapy at 125 mg BID or daily.

The initial response to vancomycin therapy was demonstrated for all 6 patients at early follow-up ranging from 1 to 3 months. All had improvement of clinical symptoms with reduction in stool frequency and improvement in consistency. Some had bloody stools, nocturnal symptoms, and abdominal pain, which also improved.

One patient is awaiting post-vancomycin endoscopy. The remaining 5 started with moderate-to-severe inflammation and improved to mild or no inflammation on follow-up endoscopy 2 months to 2 years after starting vancomycin. Two patients had documented repeat endoscopy at a longer period of follow-up (4 and 6 years) and maintained remission.

The follow-up duration ranged from 2 months to 6 years. No adverse effects to vancomycin were reported. None of the 6 patients required steroids, addition of alternative therapy, admission, or surgery.

All patients were on concurrent therapy (vedolizumab, ustekinumab, or 5-ASA) either started prior to or at the same time as vancomycin. Three of these patients discontinued their ustekinumab at 6, 8, and 14 months respectively given treatment effectiveness and maintained remission on vancomycin alone.

**Conclusions:**

In a subset of patients with PSC-IBD, vancomycin can induce clinical and mucosal remission for refractory IBD. We present two dosing regimens which produced rapid clinical improvement and those with longer follow-up sustained durable results. Prospective studies with larger sample sizes are needed.

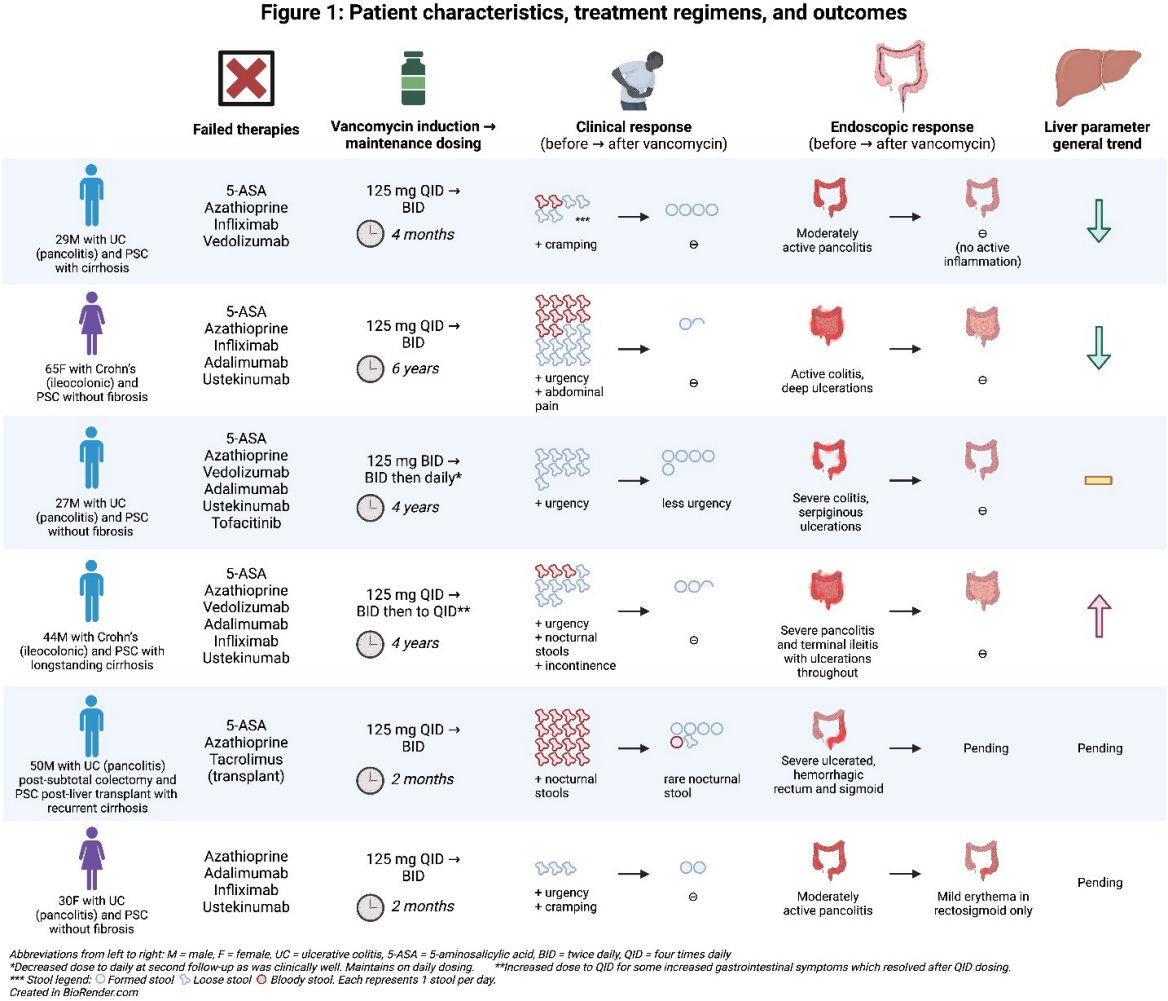

**Funding Agencies:**

None

